# Legitimizing strategies in transformative spaces: institutional meaning-making and organizational change in health care

**DOI:** 10.1108/JHOM-01-2025-0054

**Published:** 2025-09-05

**Authors:** Hanna Komulainen, Nina Lunkka, Noora Jansson, Merja Meriläinen, Heikki Wiik, Marjo Suhonen

**Affiliations:** Faculty of Social Sciences, University of Lapland, Rovaniemi, Finland; Wellbeing Services County of North Ostrobothnia, Oulu, Finland; Department of Health and Social Management, University of Eastern Finland, Kuopio, Finland; Research, Development and Innovation Unit, Oulu University of Applied Sciences, Oulu, Finland; University of Oulu, Oulu, Finland

**Keywords:** Organizational change, Institutional context, Critical discourse analysis, Legitimization, Health care organization, Transformative space

## Abstract

**Purpose:**

Our aim is to reveal how the institutional context and the surrounding social practices can shape people’s meaning-making processes in transformative spaces through discursive legitimization practices.

**Design/methodology/approach:**

We employed a case study approach and analysed ten video-taped hospital development meetings, using Fairclough’s critical discourse analysis.

**Findings:**

According to the analysis, societal, managerial and professional discourses and discursive legitimization strategies of rationalization, normalization, authorization, narrativization and moralization collectively played a major role in the transformative space negotiations in legitimizing the content and means of change in the health care organization under study. These strategies allow us to pay attention to whichever voice is heard loudest and most shapes the reality in a transformative space.

**Originality/value:**

While previous studies have acknowledged the inclusive and interactive nature of transformative spaces, far less attention has been given to the influence of institutional context on the negotiations occurring in these spaces.

## Introduction

Health care organizations operate in an ever-changing environment; they seek to combine financial sustainability and customer focus (Cosenz *et al.*, 2024), while navigating the constantly unfolding impact of political decisions and demographic changes (Erhola *et al.*, 2020). Thus, continual change processes are integral parts of health care organizations (Persson, 2014). Traditionally, changes in health care organizations are seen as top-down processes that are led by rationalistic management (Choi *et al.*, 2011). Such a view tends to ignore the importance of staff’s participation in change processes, which may, e.g. increase resistance to change (Amarantou *et al.*, 2018). Some studies argue that this may be one reason for the markedly poor success of change initiatives in health care settings (Erlingsdottir *et al.*, 2018).

Considering the foregoing therefore, recent studies have started to highlight the bottom-up nature of organizational change processes in health care (Valleala *et al.*, 2015; Komulainen *et al.*, 2019). Furthermore, some studies suggest that inclusive spaces may possess transformative potential in the health care setting. Transformative spaces are created by management to enable collective meaning-making by staff and management together, the aim being to foster positive change and transformation (Kellogg, 2009; Bucher and Langley, 2016; Lunkka *et al.*, 2022). However, despite best intentions such meaning-making may be partial, one-sided or biased, as people in transformative spaces make sense of what is occurring based on their personal experiences of deep-rooted patterns of thought, assumptions and interpretations (Weick, 1995).

One important aspect related to this is the (normally) highly institutionalized health care system – we are referring to taken-for-granted structures, practices and meanings – that tends to shape what people within their jurisdictions think or aspire to (Reay *et al.*, 2016). This marked degree of institutionalization often hinders change and transformation in health care organizations (Reay *et al.*, 2016). Yet little attention has been given to the influence of this broader setting on meaning-making processes in transformative spaces. There is scant existing research on naturally occurring change negotiations in transformative spaces; the prevailing view sees the institutional context simply as the ongoing accomplishment of actors (Lunkka *et al.*, 2022) rather than as an influential element in negotiations. As such, the focus is on investigating how people themselves produce and maintain institutional structures through everyday micro-level meaning-making; the larger socio-political and historical contexts that shape their meaning-making processes have thus far been largely ignored in the literature.

We address this by problematizing such micro-level views and aim to reveal how the influence of institutional context and the surrounding social practices significantly impacts on actors’ meaning-making processes in transformative spaces. By using Fairclough’s *et al.* (1997), Fairclough’s (2003) critical discourse analysis (CDA), we specifically focus on discursive legitimization strategies through which actors in transformative spaces legitimize events and actions, or their wider acceptability (Vaara *et al.*, 2006; Fairclough, 2003). Discursive legitimization strategies provide an excellent route to reveal which institutional-level structures frame reality when making sense of change (Jørgensen and Phillips, 2002). The objective of this paper then, is to identify the institutional-level discourses that shape legitimation strategies in transformative spaces and declare how they construct and reinforce certain worldviews and marginalize others. The research question underpinning this is: *How does the institutionalized health care context shape discursive legitimizing strategies in transformative spaces?* The deeper motive of the paper is to help health care practitioners become more conscious of the pervasive influence of the institutionalized health care setting in the transformative spaces that they inhabit. Improving this sense of awareness will elucidate the fact that meaning-making is not limited solely to the characteristics or skills of the individual practitioner; meaning-making is also influenced by broader discursive and social practices embedded in institutionalized health care systems: these also need to be considered much more than they have been to date.

Our research took place in Finland, where health care organizations are politically driven professional bureaucracies. These organizations are publicly funded and therefore subject to many conflicts of interest. The service delivery structures consist of both the internal division of labour and the division of labour between other health care organizations and actors in the wider health care system (Kokkinen, 2012). Empirically, we investigated video-recorded work development meetings in a university hospital and analysed managers’ and health care professionals’ meaning-making by focusing on their naturally occurring language use. The meetings were used by the management for planning and discussing collectively with the staff a wide range of changes thought necessary for improving the functioning of the unit.

The study provides the following main contributions. First, our critical study of naturally occurring change negotiations in collaborative health care change processes (Mikkola and Stormi, 2021; Komulainen *et al.*, 2019), particularly in transformative spaces (Lunkka *et al.*, 2022), enables a detailed examination of meaning-making processes in a manner that takes into account the profound influence of the institutional context on such processes. The practitioners’ language use manifests legitimizing strategies that were mainly shaped by societal and managerial discourses at the expense of professional discourse, thus promoting certain interests over others. Second, our study extends the existing research on the powerful potential of transformative spaces (Kellogg, 2009; Bucher and Langley, 2016; Lunkka *et al.*, 2022) by illuminating how the highly institutionalized health care system, through discursive practices, influences the course of change by limiting opportunities for alternative interpretations or critical reflection about change. In this way, our study brings to light how the institutionalized health care system may hinder the change potential of collaborative and inclusive spaces; as a result, there is a danger that these spaces may rather produce stability than change. However, our study also demonstrates where the possibilities for change may lie by identifying the moments of delegitimization that may fracture the prevailing discursive order.

The paper proceeds as follows. The following sections set out the theoretical framework of the study, describe the research methods and findings, and discuss their implications.

## Legitimizing change in transformative spaces in health care

### From change management to transformative spaces

Organizational change processes in the health care setting have been well addressed in previous literature. Traditionally, they are considered as top-down, straightforward, phased and linear processes that are merely a managerial issue (Salmela and Fagerström, 2008; Chreim *et al.*, 2012). Such an approach to change processes has provided valuable practical knowledge, e.g. on change management, and identified several important factors that are worth noting in the implementation of change (Demers, 2007). However, this view tends to ignore the way in which change is collectively negotiated by different actors in health care. It has been argued that this is one reason why change processes in health care often fail (Erlingsdottir *et al.*, 2018). In addition, the traditional approach has been criticized for being overly rationalistic, typically omitting the everyday environment within which change processes happen (Choi *et al.*, 2011; Erlingsdottir *et al.*, 2018).

An alternative approach to studying organizational change processes in health care focuses on an interpretation of reality and micro-level meaning-making processes. This approach often adopts a discursive or narrative view of change processes, where change is thought to be socially constructed through personal interactions (Mikkola and Stormi, 2021; Komulainen *et al.*, 2019). This approach has extended the traditional approach to studying change processes, highlighting the fact that change requires a shift in the mindset and meanings that drive the activities of the actors in an organization (Demers, 2007).

To this end, participative and collaborative spaces may have transformative potential by enabling inclusion and collective meaning-making about the situation apart from regular work activities, thereby promoting change (Lunkka *et al.*, 2022; Bucher and Langley, 2016; Kellogg, 2009). Transformative spaces can be used to motivate health care professionals to jointly develop, evaluate, and question existing work practices, e.g. by using context-bound practical reasoning (Lunkka *et al.*, 2022). Transformative spaces are in this article considered as small-scale settings set up by management, which provide an opportunity to discuss, make sense of and legitimate aspects of the change process collectively and outside of normal work arrangements (Lunkka *et al.*, 2022; Bucher and Langley, 2016; Kellogg, 2009).

While recent studies of transformative spaces have emphasized their inclusive and transformative nature (because they allow collective negotiations about a situation), researchers have nevertheless seldom focused on analysing the influence of the highly institutionalized health care setting on these collective negotiations. The existing research on naturally occurring change negotiations treats the institutional context essentially as the ongoing result of everyday interactions (Lunkka *et al.*, 2022), ignoring how it constrains and shapes meaning-making. Yet the institutional context of health care is known to often complicate, and hamper change efforts in health care organizations (Nielsen and Riiskjær, 2013; Reay *et al.*, 2016). Even when there is significant new knowledge to support the renewal and transformation of health care operations and services, it is sometimes difficult to get health care professionals to think differently and enact change in practice (Gkeredakis *et al.*, 2011). In addition, the professionalized nature of health care means that traditionally conservative professions have an innate capability to either promote change or maintain stability in the system. Although stability in the system is important to the reliability and predictability of high-quality services, it also tends to prevent beneficial changes in health care organizations (Reay *et al.*, 2016). For these reasons, it is important to elaborate our understanding of transformative spaces in health care in a manner that acknowledges the influence of the broader – and often restrictive – institutional context of health care.

In this article, we seek to enhance understanding of the meaning-making processes in transformative spaces by examining the legitimization strategies used *in situ* by different actors while negotiating changes to the unit’s activities.

### Capturing the institutional context of transformative spaces through legitimization strategies

In transformative spaces, isolation contributes to the construction of the legitimacy of new working models (Kellogg, 2009) and spaces, conveying ideologies that privilege some meanings over others (McDonald *et al.*, 2017). Our goal is to understand the processes that are at work here. Legitimization strategies are discursive practices, i.e. conventional ways of using language. They act as mediators between language use in a transformative space and a health care organization’s institutional context, creating a relationship in which socio-cultural practices influence the nature of legitimization strategies. (Fairclough *et al.*, 1997) The institutional context acts as an external power, limiting or supporting social actors in transformative spaces. It defines norms, rules, values and participants in events, and determines what topics and goals are pursued, and thus facilitates or constrains people’s meaning-making processes in transformative spaces (Fairclough, 2013).

Hence, a deep understanding of the institutionalized health care system becomes necessary when investigating discursive legitimization strategies in transformative spaces. Health care organizations undergoing change processes have been found to integrate elements of both New Public Management (NPM) and New Public Governance (NPG), the former emphasizing efficiency-driven managerialism and the latter collaborative approaches (Tenbensel *et al.*, 2021). NPG emerged as a response to both hierarchical forms of control and the market-driven practices strongly associated with NPM. NPG embodies a networked and multi-actor mode of governance, where service delivery and problem-solving are grounded in collaboration and stakeholder participation (Helgesen, 2014). At its core, NPG emphasizes the creation and negotiation of values, meanings and relationships (Osborne, 2010).

Although collaborative aspirations are currently gaining traction, NPM practices remain deeply embedded in health care settings (Tenbensel *et al.*, 2021). Activities of health care organizations are guided by the politically supported management practice of NPM, which mainly focuses on resource discipline and cost-effectiveness (Nordstrand Berg and Byrkjeloft, 2014). Health care organizations are bureaucratic professional organizations that may face challenges such as structural rigidity, lack of patient orientation and overall inefficiency. In addition to top-down management, hierarchical relationships between professional groups also exist within the organization (Kokkinen, 2012). The activities of professionals in health care organizations are regulated by the government through legislation, which covers many different perspectives on the services provided. Government action is based on anticipating and responding to societal changes through policy decisions that guide organizations (Ministry of Social affairs and Health, 2022).

It is also well known that the health care context is characterized by asymmetrical professional knowledge, professional hierarchy and professional norms (Currie *et al.*, 2012; Nielsen and Riiskjær, 2013; Reay *et al.*, 2016). Physicians, nurses and managers are the most prominent professional groups in health care organizations (Currie *et al.*, 2012; Nielsen and Riiskjær, 2013). The perspectives of nursing staff and physicians focus on patient-centredness and professional values (Evetts, 2011), while managers maintain efficiency thinking and resource discipline (Nordstrand Berg and Byrkjeloft, 2014). Professions play an important role in health care, as they can simultaneously create, legitimize and control the knowledge and practices that organize and guide different aspects of everyday working life (Scott, 2008b).

## The study

### Research setting

The research setting was a university hospital in Finland. At the time that the study was conducted, specialized health care was publicly funded and supported through discrete hospital districts (21 in total), which were either joint ventures undertaken between municipal authorities or collaborative entities owned by municipalities (Finlex 1062, 1989). The Ministry of Social Affairs and Health was – and still is – responsible for national governance, legislation and strategies (Ministry of Social Affairs and Health, 2022). Statutory regulation included, among other things, the care guarantee law, national legislation on staffing requirements, and a (then forthcoming) national decree that was expected to affect the division of labour in specialized health care as well as the centralization of certain medical examinations, procedures and treatments. Hospitals are intriguing organizations for analysing transformative spaces within the wider health care system because their activities are heavily politically regulated and influenced by several institutionalized stakeholders (Nordstrand Berg and Byrkjeloft, 2014), and there are intricate divisions of labour due to stratification and professional hierarchy (Bucher *et al.*, 2016). In addition, governments and health care organizations are working hard to improve patient care quality and the efficacy of health services by supporting multi-professional collaboration (Kellogg, 2009; Zamboni *et al.*, 2020). It is obvious that these demands raise the need for collaborative change processes that support the negotiation and creation of desirable new work practices (Cregård, 2018).

Our case study was a specialized care unit that had faced increasing strain due to rising patient numbers and the growing complexity of care. Earlier efforts at innovation in 2006 did not lead to lasting change, and since then, the increasing number of patients has led to difficulties in providing timely care, and staffing levels have proven insufficient to meet the growing demand. Nursing professionals reported challenges related to competence management and workload. In addition to all of this, a forthcoming organizational change due to a move to new premises and the effects of national health policy reforms were also expected to further increase pressure. To address these challenges, a series of multi-disciplinary development meetings was held, functioning as transformative spaces where participants collaboratively negotiated over issues such as operations management, work planning, competence development, patient pathways and logistical systems.

### Research material

The research material was collected before the major social and health care reform in Finland came into effect in 2023. The research material consists of 20 h of video recordings comprising ten two-hour development meetings held between August 2018 and March 2019, covering the full seven-month collective planning phase of the change process. This extensive body of audio-visual material provides a focused lens on the discursive construction of institutional meaning-making in transformative spaces. The meetings were organized separately from daily work and involved professionals from different specialisms. They focused on improving the situation in the unit and on constructing legitimacy and meanings for change. Therefore, these meetings could be thought of as transformative spaces (Kellogg, 2009; Bucher and Langley, 2016). The development meetings were a form of collaborative work convened by the unit’s management to develop the unit’s crisis operations in a multi-professional manner. The meetings were led by an external consultant (none of the authors of this paper) who designed and facilitated and organized the meetings.

A total of 40 people participated in the ten development meetings, including managers from different organizational levels, surgeons from various specialties and nursing staff with diverse roles. The participant group remained largely consistent, with only minor substitutions within some of the professional groups. This diversity of expertise enabled a longitudinal analysis of institutional meaning-making while maintaining continuity in perspectives and roles. Meetings were organized once or twice a month and were video recorded by the external consultant. Context-bound recordings made it possible to analyse the language use in the meetings. Thus, it can be concluded that the method provided a good basic understanding of the phenomenon and structures under study and allowed the identification of the institutional contextual factors that lay behind the legitimacy speech (Alvesson and Sköldberg, 2018).

Prior to collecting the data, research permits had been applied for by the hospital organization in question. Participants were informed about the use of video material for scientific research in connection with the work development meetings, and they gave their informed consent when attending the meetings (Denzin and Lincoln, 2011). The anonymity of participants was ensured in the research process. No names or professional specialisms have been published in the results. As such, an individual participant cannot be identified from the results presented in the study (Bell and Wray-Bliss, 2010). The first author was working as a health care professional in specialized care. Thorough familiarity with the specialized health care practices brought greater depth to the analysis, which helps with understanding the contextual framework and improves the accuracy of our interpretation. The researcher’s professional position provided an invaluable degree of sensitivity and consequent insight into how meanings were constructed and experienced by actors, as she was able to identify and pinpoint subtle nuances, tacit knowledge, and underlying assumptions that might not have been apparent to an outsider. On the other hand, of course, we acknowledge that the professional background of the first researcher, covering both practitioner and managerial roles, may have coloured her interpretation of the results. Although the analysis was able to draw on a wide range of perspectives based on extensive professional experience, it is possible that certain conceptual assumptions remained implicit due to entrenched patterns of thinking. However, engaging in collective reflection with the rest of the research team helped to develop and sustain a sharply honed critical perspective, ensuring that phenomena potentially taken for granted by an insider were also brought into analytical focus. During the study, constant critical self-assessment and engagement with the research material were maintained to keep the focus on the subject, with the researchers’ position serving solely to support interpretations. From a methodological point of view, the use of video recordings as research material increased the credibility of the findings, as it allowed for observation of the natural conversation *in situ*, and there was always the possibility to return to the recordings for repeated viewings.

## Analysis

The video recordings of the meetings were transcribed for textual analysis. The analysis focused on the discursive practices of legitimization, i.e. the use of naturally occurring language in the transformative space, so facial gestures and body language were not included in the analysis and no additional research material (e.g. interviews) was collected for the study. No software was used in the analysis, as the focus was on the meaning-making processes which developed through legitimization strategies – i.e. on the interpretation of ways of speaking (Jørgensen and Phillips, 2002) – rather than on the frequency of specific words or discourses. To analyse language use to illuminate the institutional meaning-making processes, we adopted Fairclough *et al.* (1997), Fairclough’s (2003, 2010), where discourses are understood as socially constructed, established perceptions of reality, with characteristics that have been shaped by the institutional context (Fairclough *et al.*, 1997, Fairclough, 2003).

Conceptually, following Jørgensen and Phillips (2002), we thought of the transformative space as a communicative event in which strategies of legitimization were involved in maintaining a social world, including unequal power relations. Legitimization strategies were part of the institutional context surrounding the transformative space and acted as a mediator of the institutional context’s perspectives in the negotiations. These perspectives were the ones by which actors make sense of the change process. The analysis of legitimization strategies focused on how speakers used existing discourses to create legitimacy. It meant that we identified how actors in transformative spaces represent different discourses, and which discursive legitimization practices involved which actors and in what combinations in those negotiations within this context. Discourses were context-bound, they provided a basis for legitimacy, and their identification helps to understand the order of discourses and interdiscursive relationships (Fairclough, 2003). The order of discourses refers to the entire system of multiple discourses operating within the context of the hospital’s transformative space (Jørgensen and Phillips, 2002). Together with interdiscursivity, it illustrates how participants in conversations were oriented to represent and identify needs and means for change through the discourses they maintained and how they positioned themselves in conversations. The order of discourse implied a hierarchy and a power perspective of dominant discourse in the transformative space: some discourses were more dominant, while others received less attention (Fairclough, 2010).

As an analytical idea, the basic premise was that change could be promoted by combining discourses in new or otherwise unconventional ways compared to what usually happens in the typical context of health care. Such innovative discourse combinations may open up possibilities for change and for challenging the dominant ways of legitimizing it. On the other hand, more traditional ways of expressing and combining discourses contributed to continuity and reinforced existing power relations (Jørgensen and Phillips, 2002). For example, in change negotiations, managerial discourse could incorporate elements of both employee well-being and service effectiveness, forming a novel interdiscursive combination. In contrast, when aiming to maintain continuity, change may be legitimized through more traditional references to efficiency and cost-effectiveness.

Following Vaara *et al.* (2006), legitimization is thought to be a discursively created sense of what is thought to be legitimate or delegitimate. Legitimizing strategies reflect the institutional context of the organization, highlighting generally accepted and maintained rules, norms, and practices (Vaara *et al.*, 2006; Scott, 2008a). Legitimacy is intricately linked to the institutionalization of social phenomena (Suchman, 1995), so in the context of legitimating change, it means the legitimacy of broader cultural and societal beliefs and values, but also the power position of managers (Deephouse and Suchman, 2008). The strategies of legitimization followed the ideas of Van Leeuwen and Wodak (1999) and Vaara *et al.* (2006): rationalization, normalization, authorization, moralization and narrativization.

Rationalization in this case refers to the legitimization of the change or means of change, highlighting the usefulness or operation of certain actions or practices in a certain way, and the need to streamline the operation, in this case considering the nature of the hospital/unit’s activities and the imminent new building (van Leeuwen and Wodak, 1999). Rationalization and moralization are closely related because rationalization is often based on value-based perspectives. Normalization seeks to legitimize the process of change by exemplary actions or by making the phenomenon seem normal by comparing it with similar cases and activities in the past or with expected future activities/events. (Vaara *et al.*, 2006). Authorization is legitimization by explicit reference to authority. Means of authorization include appeals to tradition, custom, law, rules, guidelines and influential people as a higher authority (Van Leeuwen and Wodak, 1999). The legitimacy of moralization is always based on values. The values of moralization are, in many cases, subtly referred to by certain loaded adjectives. For example, adjectives such as are *healthy*, *fair* and *useful* all have an intrinsic reference to good morals (Van Leeuwen and Wodak, 1999). Moralization is also used to delegitimize, which is an attempt to show the inaction of an action (Vaara *et al.*, 2006). Narrativization brings to light the substantive and descriptive legitimization for the process of change. This refers to Van Leeuwen and Wodak’s (1999) idea of the mythopoetic side of legitimization, of how telling a story provides evidence of acceptable, appropriate or preferential actions. But Fairclough (2003) also presented the idea that mythopoesis can additionally involve some forms that are not narratives in their strictest pre-genre or genre sense.

The analysis followed Fairclough’s three-dimensional model (Jørgensen and Phillips, 2002). The coding of legitimation strategies was theory-driven, firmly based on the research question and also on previous frameworks of legitimation (van Leeuwen and Wodak, 1999). The process involved repeated rounds of coding and interpretation in which the language use in the transformative space was compared with the theoretical framework. To increase analytical validity, discussions were held with other researchers. In addition, this study included a reflective break, which enabled the researchers to increase their understanding of the topic and then revisit the coding, feeling intellectually refreshed after the break.

The analysis started with us getting fully acquainted with the material. First, a thematic analysis was conducted, as recommended by some critically oriented researchers (Vaara *et al.*, 2006). This provides an understanding of the themes of the negotiation within which the changes and means of change were discussed and legitimized. The themes on which legitimization was based in the negotiations were: hospital organizational and societal factors, staff well-being and competence, operational perspectives, patients and resources. The themes were strongly interlinked in the negotiations, and often one thing led to another, which well reflects the diversity, complexity, multiple levels of collaboration and multi-obligation of hospital phenomena. The research material was read through several times to get a sense of the whole: the vocabulary and verbal expression of things shaped the main discourses of the transformative space. Here, particular attention was paid to which issues were discussed and how they were discussed. After this, we selected extracts illustrating the legitimacy of the change process for further analysis and identified strategies that were used in accordance with the description of the legitimization strategies outlined above. There were three main discourses discerned as present in the transformative space: *managerial*, *societal* and *professional*.

It transpired that all five strategies were used, and it was also noticeable that, e.g. rationalization appeared in all three discourses; thus, the legitimization of change came from slightly different starting premises. At this point, we were interested in what kind of reality the legitimization strategies created, maintained or questioned within these discourses and how the participants positioned themselves when legitimizing the change process. Close textual analysis covered vocabulary (naming things, categories, recurring words and terms), grammar (modality, use of adjectives and adverbs), and syntax (blurring of the actor in transitive and parallelism/subordination) (Jørgensen and Phillips, 2002). Lastly, the findings were refined and placed in a broader context (including analysis of the order of discourse and what consequences this had for broader social practice). The first author conducted the analysis, and the others commented and participated in the discussion during the analysis when needed.

## Discursive legitimization strategies in the transformative space

Below is a presentation of the legitimization strategies used in the transformative space, categorized by discourse. Negotiations took place in a specifically transformative space as the people present explained why the meetings had been convened. The participants in the transformative space were introduced to the overall situation of the unit, and in working groups they planned different types of operational changes, such as: competence-based team distribution of nursing staff, orientation of the ward attendant, a sustainable approach to the operation of the unit, including the opening of an additional on-call room, improvement of storage and logistics, and development of the recovery room. Several different discourses were present in the transformative space’s negotiations, of which the managerial, professional and societal discourses stand out most clearly here.

### Managerial discourse

Managerial discourse combined the efforts of organizational actors to stick to the long-term objectives and strategic decisions of the organization and reflected the efficiency and accountability of NPM. Also included were competence management and well-being at work, as professionals are seen as a strategic resource to adapt to a changing environment and achieve organizational goals. The positioning of the actors as financial controllers or the intermediary message importers supported NPM perspectives and sought to strengthen its message or the position of the managerial discourse in the organization, e.g. *“Matters relating to employment relationships in the hospital district are unfortunately rigid and regulated by administrative regulations in such a way that even the director of the performance area is unable to intervene in the matter.”* This sentence has elements of transitivity, which means that the decision-makers of administrative regulations are obscured, which in turn confirms that administrative regulations cannot be influenced.

*Rationalization* within this discourse referred to the strategic decisions of the organization, i.e. the new building and the need for operational changes related to future competence centres and well-being at work. It is worth noting that two of these were prospects for the future and the fact that the unit was already facing challenges in sustaining its operations, so from a rationalization perspective, they gave a strong signal of the legitimacy of the change processes. Rationalization supported the organization’s strategic choices and promoted its adaptability to environmental and societal demands. It created the taken-for-granted reality that strategic decisions take change in a particular direction. The economic perspective was not as clear in legitimizing change as, e.g. in Vaara *et al.* (2006), but it manifested itself as resource discipline and an effort to cope with existing limited resources. This was most evident in the close connection between rationalization and moralization when the world of professional values was service- and customer-centric. The managerial discourse included the resource discipline of NPM, which created the basic reality that there was to be no substantial increase in staff, even though the number of patients would increase in the future due to changes in the age distribution of the population.

As the crisis in the unit was affected by staff coping problems, the need for change was also rationalized from competence management and well-being-related perspectives. This was used to create a reality that work *had* to be evaluated and changed to maintain job satisfaction. It was noteworthy that the means of change were directed at being a reassessment of activities, not an increase in resources. The examples also showed that it is impossible for everyone to know everything. In this context, competence-based team structures were seen as one solution to the problem of coping. In addition, the competence centres that were planned with the new building led to a strategic decision by the organization that it would make sense to divide the nursing staff into teams according to their competencies. In this case, too, the legitimacy created by the strategic decisions made within this organization was assumed to be taken for granted.

The language use of transformative space had several modal structures (Vaara, 2010), which strengthened the governance of the rationalization strategy (*“I don’t think there’s any reason*; *you can’t imagine*; *it is not possible to know everything*; *you can’t think*; *you must find a new model …”*). For example, Table 1 resource discipline (*“We should not imagine that our staff numbers would increase substantially … ”*) was presented as an absolute outlook for the organization’s human resource management; the absoluteness was emphasized by the continuation of the sentence quoted above that this would be the case even if patient numbers increased.

**Table 1:** The construction of legitimacy in managerial discourse

Legitimization strategy	Meaning-making themes	Examples
rationalization	strategic decisions	*“When you think about these things and think that how to organize this activity from now on, so that it will last then, to go to new building.”*
		*“I don’t think there’s any reason to imagine that, going into the new environment, with the current approaches. There’s an opportunity to do things differently and there’s a reason to do things very differently ….”*
		*“I think this development project has come at an excellent time, and this is one of the components in the design of the new hospital.”*
	resource discipline	*“We shouldn’t imagine that we’re going to have a substantial increase in staff numbers, even though our age group, the old-age group, the over-seventies, they’re going to almost double by the year -30.”*
		*“If we add teams in the evening, we should get more of those job vacancy with which we are in trouble anyway at the moment.”*
		*“The truth is that we cannot increase the number of employees at the moment, so we should kind of by Leaning to see where we could get the resource there for the nights”*
	competence management	*“There’s been talk about these competence centres for the new hospital, it’s quite a big change in concept. So if things like this are going to happen there, it would probably be worth taking them into account in the division of labor that is being planned here …”*
		*“These competence centers have been thought up for new premises … The one direction of solution is that it is necessary to get smaller domains in order to, competence can be managed because, we talked about the fact that there will be more and more new types of surgery, surgical methods, anesthesia forms and so on.”*
	well-being at work	*“We are constantly in a race to survive and to be able to cope, and I have understood that the unit has come to the end of the road in the ‘back skinning’ … You must find, in every place where you work, a new model that saves the backbone.”*
		*“That’s why there have been so many challenges in coping, and actually the remedy is to develop the activity, so that I can make it smoother, so that I can do it, and be able to succeed in the work, in the job, whether I’m a doctor or a nurse or any other professional or manager.”*
normalization	successful development in the past	*“I remember that the working model at the time, for example, reduced sick leave for nurses by 30% the following year, so yes, it did matter.”*
narrativization	experiential message from a larger team	“*I mean, the same kind of message you hear now is that you don’t have energy and there’s a lack of work management and lack of skills. And from the surgeons there is a bit of the same message, that is how their own team can’t really get together and can’t function well enough.”*
	visionary narrative	*“There is a general feeling that things are not under control, and on the other hand, we also know that operations have grown since 2006 … Maybe now, with the bigger size, it would be easier to find a better model to manage this, to manage skills, to make the staff able to cope better and to improve their well-being at work.”*

**Source(s):** Authors’ own work

*Normalization* referred to the work development that had taken place in the unit in the past and the benefits it had brought, particularly in terms of staff well-being (Table 1). This created a reality that similar work development had taken place in the past and had improved the competence situation and well-being at work. At the textual level, the impact of the earlier change was reinforced by informing those present with quantitative information on the reduction in sick leave. The idea was that if the last change was successful, then this one had a chance to be successful too.

*Narrativization* pointed out that there was an awareness of the team’s experience, workload and the growth of the unit. There was an attempt to create a common understanding and sense of community around the need for change. This allowed the change to be legitimized by presenting it as a solution to the team’s problems and highlighting the opportunities for growth and development. At the same time, it created a sense of inclusion and participation that could support acceptance of change and reduce potential resistance. The narratives had a motivational and encouraging tone, as this creation of a competency-based approach was one of the major changes being planned and was discussed in the majority of the ten meetings.

### Professional discourse

This discourse maintained professional values and patient-centredness. In this discourse, actors positioned themselves as upholders of ethical standards and custodians of patient well-being. This discourse was central to professional identity and the shared practices that guided daily work and legitimized professional practice at different levels. It produced a sense in which actors perceived their responsibility for patient well-being as part of a larger, collective goal.

*Rationalization* within this discourse was based on the fact that medical and nursing techniques and methods of treating patients have developed and will continue to do so, making it possible to maintain and improve the quality of care, treat sicker patients and carry out more advanced surgeries. This has had – and will continue to have – an impact on both patient numbers and procedure times. Rationalization was the motive for the need for change. This provided a strong legitimization for the change, because as a university hospital, the organization was also at the forefront of developing both medical and treatment methods.

*Moralization* was based on different perspectives on patient access to care. As patient numbers and operating hours had increased, emergency patients were often left on the wards waiting for surgery. This was a concern for professionals, and one of the reasons why common rules were not always respected. The concern was combined with the idea of timely care for patients and increasing workloads in in-patient wards. The build-up of patients in the wards meant that others sometimes had to be left in the corridor. Legitimization of the strategy was based on professionals' patient-centred values. The reality created by the strategy highlighted the fact that an increase in resources during daytime hours would enable faster patient throughput, ensuring they do not become a burden at any stage of the process.

The moralization strategy was also used for delegitimization in our study, as in Vaara *et al.* (2006). Delegitimization reflected values that differ significantly from NPM resource discipline, rigid human resource management and the implementation of legislative decisions. There was a strong statement against the organization’s rigid human resources management in Table 2, but actors in this space said that unfortunately this was beyond their control (see an example from the beginning of the section on the managerial discourse). This confirmed the strength of NPM as a driver of change. The response protected the organization’s governance regulations and maintained its strong legitimacy within the organization.

**Table 2:** The construction of legitimacy in professional discourse

Legitimization strategy	Meaning-making themes	Examples
rationalization	developing techniques and methods	*“The need for development is that the methods of surgery, anesthesia methods, and technology are developing so that you can treat more and more demanding patients, and it starts to show up in the numbers and in the hours of surgery.”*
moralization	patient access to care	“*Those patients who could do it that day are an extra day in the ward … Now those same patients are waiting day and night, in some ward, for access to surgery.”*
		*“When the care guarantee is about to expire and the fact is that someone could sue me in court, when they already go, I’ve put two of them in there, I do tricks, and I know when I put these on the surgery list that these are not going to happen.”*
	delegitimization	*“The first thing is people’s employment relationships. We know that this, the work, is not going to end. The amount of work will increase and when we go to the new organization, the need for staff will not decrease. I heard that they were looking for three new nurses for this on-call team, if the application offers a job for three months, I’m not terribly surprised if there are no applicants from far and wide … This is like a blessing that must come from the house level that something must change in basic policy*
		*“The centralization thing, if it is coming, is it just so that they just come, that we are not asked at all, to say that we do not have facilities ready here … ”*
		*“At some point we have to think about getting additional resources, because we haven’t found any good ideas from any direction without additional resources.”*

**Source(s):** Authors’ own work

### Societal discourse

In the societal discourse, the public hospital was presented as part of a wider social and global system. It sought to highlight the need for the hospital to adapt to global trends and societal pressures while striving to ensure equity and efficiency of care. Societal discourse was the most normative in nature and maintained the views that were in line with the goals of the welfare state. These reinforced the authorization strategy and the message of the inevitability of change brought about by political decisions.

*Rationalization* within this discourse included the idea of the global demographic ageing trend, which was inevitable and beyond anyone’s influence. This was raised in Table 3 – what kind of reality this inescapable fact shapes in the health care organization. There were elements of modality in the form of truth in that extract related to the inadequacy of emergency operations. The number of patients has increased and will continue to increase. Everyone knew that demographic changes were inevitable and would put further external pressure on the organization to make more efficient use of resources and maintain its resilience. This was a strong argument legitimizing the need for change.

**Table 3:** The construction of legitimacy in societal discourse

Legitimization strategy	Meaning-making themes	Examples
rationalization	global demographic aging trend	*“The number of operating hours has skyrocketed. And now, in the last two years, the number of on-call hours has also increased by 13%*
		*“The number of emergency patients is increasing all the time and the number of elective patients is increasing because we are also increasing the number of referrals by about 10% every year at the moment.”*
authorization	legislation	*“There will be more patients on the horizon and there will be centralization and if other hospitals do things like that here, there may be more orthopedic and gastro patients and so on. So, the need for this will not diminish.”*
		*“I wonder how these centralization regulations and others then again, and soon there will be a lot of patients.”*

**Source(s):** Authors’ own work

*Authorization* in the transformative space was legislation defining the activities of the public hospital. The government decree on the division of labour in special medical care and the centralization of certain tasks (Finlex 582, 2017) was a known new change in legislation that would increase the number of patients in the unit soon. Although the outcome of the change in the law was not yet known, it was included in the negotiations, reinforcing the need for change, but also increasing uncertainty and the diversity of change. This strategy created the reality that the forthcoming change in the law would bring absolute changes to the functioning of the unit in many ways. In this discourse, there were absolute structures that reinforced the authorization strategy and the message of the inevitability of change brought about by political decisions.

## The interrelationship between the legitimization strategies and the health care context

Next, we looked at the institutional context of health care, of which the legitimization strategies were a part. The legitimization strategies used in the transformative space employed elements of managerial, professional and societal discourses as tools of legitimization. Actors in the transformative spaces maintained a reality and made sense of change and its meanings through the institutional context. Basically, in this way, the participation of multi-professional actors in the transformative space made it possible for different realities to become visible and their participation in the legitimization in connection with the change processes to emerge. However, all legitimization strategies and their contents as a means of legitimization were not equal.

In the institutional context of this health care organization, the societal and managerial discourses dominated the discourse order in the transformative space. The legitimization strategies were strongly shaped by the wider national political and economic levers present in managerial and societal discourse. In the Finnish context, specialized health care is guided by national legislation and funding models, which set the framework for the organizations' activities and use of resources. These structures produce discursive constraints within which change is legitimized; in a sense, they can be seen as politically imposed boundaries in the order of discourses. Societal discourse was the most deterministic, with political decisions and social goals determining hospital practices. This pointed to the fact that the hospital operates as part of a wider social network and must adapt to a changing environment if it is to fulfil its mission. In addition, in managerial discourse, resource discipline according to the NPM and rigid hierarchical personnel management structures were described as unchanged. The legitimacy of these discursive perspectives was assumed to guide the design of the change process as a matter of course. These were expressed as permanent structures that no one present could influence. In this way, the legitimacy of the managerial discourse of competence management and well-being at work is also overshadowed by the resource discipline of the same discourse. Permanent rigid structures made the meaning-making of change unequal and narrowed the perspectives of change planning.

The construction of legitimacy in professional discourse was overshadowed by more normative topics. One of the most notable aspects of the analysis was that the patient perspective was presented differently in different discourses. The professional discourse was concerned about the quality and timeliness of patient care and the burden on in-patient wards, while the managerial and societal discourses highlighted the increase in the number of patients and operating hours.

## Discussion

Previous research on change processes in health care organizations has identified the importance of collaboration and inclusion (Mikkola and Stormi, 2021; Komulainen *et al.*, 2019), where collective meaning-making is seen as important in gaining shared understanding (Lunkka *et al.*, 2022) and in reducing resistance to change (Amarantou *et al.*, 2018). Managers are increasingly using inclusive and collaborative spaces to facilitate positive change (Kellogg, 2009; Bucher and Langley, 2016; Lunkka *et al.*, 2022). At the same time, research has also recognized that the institutionalized health care system tends to shape health care professionals’ meaning-making (Reay *et al.*, 2016). However, previous studies on change negotiations (Mikkola and Stormi, 2021; Komulainen *et al.*, 2019), specifically looking at naturally occurring negotiations in transformative spaces (Lunkka *et al.*, 2022), have focused on investigating meaning-making from the bottom-up without considering the influence of the historically formed, institutionalized health care context. The present research therefore focused on revealing how the institutional context and its surrounding social practices shaped participating actors’ meaning-making processes in transformative spaces.

Thus, our study’s first unique contribution lies in its novel, critical examination of naturally occurring change negotiations. The paper illuminated how a critical perspective on meaning-making processes in transformative spaces moves beyond situational interaction processes and provides insights into the inter-relationships between legitimation strategies and the broader health care context through which social reality was created. Using CDA (Fairclough *et al.*, 1997; Fairclough, 2003), we were able to identify the institutional-level discourses that shaped legitimation strategies in the transformative spaces and delineate their hierarchical relationships, where societal and managerial worldviews dominated and the professional (including patient-centredness) worldview was decidedly marginalized. This restricted meaning-making and critical discussions of alternative perspectives on the change process. Thus, discursive dominance narrowed the range of legitimately expressed meanings and made the voices of professionals and patients less heard in the design of the change process.

This leads to our study’s second main contribution, which centres around the potential of transformative spaces (Kellogg, 2009; Bucher and Langley, 2016; Lunkka *et al.*, 2022). The study showed that actors in the transformative space maintained in the negotiations the rigid, stable structures that guided the direction of change and limited opportunities for alternative interpretations or genuine negotiation. In this way, our research reinforced the idea of transformative spaces as mediators of ideologies (McDonald *et al.*, 2017). Notably though, only a few individual actors, through delegitimization, sought to challenge the legitimacy of the societal and managerial discourses. While health care organizations have sought to adopt collaborative aspirations, NPM practices and values are still seen as a strong collective factor in the structure and functioning of organizations (Tenbensel *et al.*, 2021). Unequal power relations may ultimately lead to a change in the value base of health care organizations and a change in their activities from specialist organizations providing good patient care, to managing simply from one day to the next with barely adequate resources. Furthermore, patient-centredness suffers when, as in this case, the patient is discussed purely from an organizational or professional perspective.

Considering the response to delegitimization, the inflexibility of governance rules may have been a sign that the organization was seeking to defend itself and legitimize its actions by sticking to rigid structures rather than confronting the root causes of delegitimization. This can lead to the fragmentation and dissolution of legitimacy rather than its construction. This may temporarily protect the organization from criticism and strengthen its control, but it can also undermine stakeholder trust if the inflexibility of the rules is perceived as an obstacle to necessary reforms or open dialogue.

In sum, the research reveals just how remarkably diverse the social reality surrounding a health care organization’s change processes is; crucially though, our work also points to where the potential for resistance and change is located (Fairclough, 2010). The organization had a good approach to engaging actors in change planning in a transformative space, which constructed ownership of the change process. By allowing multi-voiced critical discussion about the above-mentioned strongly normative reality-sustaining factors and their effects, the interactivity of the transformative space would be strengthened, and it might be possible to find alternative perspectives to construct change in this area as well. This would therefore mean that it should be possible for an organization to interpret and apply policy decisions and administrative guidelines in a way that opens new possibilities. External pressures could also be used as a tool to build alliances with other organizations to ensure the organization’s ability to operate. This had already been applied to the idea of the cooperation agreement, where the expertise available in the university hospital was to be shared with other regional hospitals (the idea was shared with the actors in the transformative space).

Supporting this form of inclusive collaboration requires a decisive shift in leadership practices towards more dialogical and collaborative governance (Tenbensel *et al.*, 2021). Managers play a key role in enabling a culture that values open dialogue, recognizes the institutional context, and invites plural perspectives into change processes. The findings suggest that genuine, solution-oriented dialogue across professional groups is possible, but it requires a safe and permissive organizational culture that reduces administrative rigidity and enhances the ability to recognize the power of discourses in collaborative negotiations during the construction of the change process. Moreover, this research may help practitioners to consider how language use and positioning in negotiations shape the meaning-making of the change process, and thus help us to understand what values, expectations, and worldviews the particular use of language upholds in the meaning-making of change. Strengthening reflexivity on these dynamics may help health care organizations resist simply reproducing the status quo and instead foster genuinely meaningful change that aligns with the values of all stakeholders, including patients. The conclusions of this study on polyphony can also be more broadly applied to the development of health care at the policy level. Strengthening and utilizing polyphony enables more effective collaboration and the efficient use of scarce human and financial resources.

The study also has certain methodological implications. Despite a few exceptions (e.g. Lunkka *et al.*, 2022), previous studies that investigated change processes in transformative spaces have mostly used interviews and field observation as data collection methods (Bucher and Langley, 2016; Kellogg, 2009). In this study, the video recordings of the transformational spaces allowed us to examine closely the language used therein (Heath and Luff, 2018). Analysing language use in transformative spaces enables the presentation of the emergent and ambiguous nature of the hospital’s change process.

In the future, one could also look at the social construction of change processes in transformative spaces through the lived experiences of the participants. Transformative spaces also provide other opportunities for further research, such as comparing the construction of reality between participants and non-participants. Video-taped meetings make it possible to study the interaction itself, e.g. through framework analysis, which would make it possible to look at what kind of multi-disciplinary interpretive frameworks the negotiations contain and how they shape the meanings of change.

We note that there are limitations to the research. The public hospital in Finland as the organization under investigation has special characteristics all its own, and this must therefore be considered when assessing the generalizability of the research results. However, the results are largely applicable to other public organizations governed by similar laws and with a comparable institutional context. In addition, health care organizations, regardless of the country or funding model, are multi-disciplinary and thus loudly polyphonic organizations, meaning that the findings of this study are certainly applicable, at least in part, to contexts outside of Finland and/or to public organizations as well. Analysing the discursive practices of the legitimization of transformative space and mirroring it to context requires careful interpretation. As stated earlier, the interpretation proffered in this study is strengthened by the professional position of the first researcher, who is thoroughly familiar with the situation in specialized health care. Finally, in the interpretation and analysis, close attention has been paid to the consistency of the research material and the results.
